# Synergistic Adsorption–Filtration of Aromatic Pollutants via Biodegradable PLA/MIL-68(Al) Mixed-Matrix Membranes

**DOI:** 10.3390/polym18101177

**Published:** 2026-05-11

**Authors:** Jiangchun Qin, Lina Dong, Hengyan Tian, Fei Yang, Jiayang Hu, Dengbang Jiang, Zhonghui Zhang

**Affiliations:** 1 National and Local Joint Engineering Research Center for Green Preparation Technology of Biobased Materials, Yunnan Minzu University, Kunming 650500, China; 2 Hubei Academy of Forestry, Wuhan 430075, China; 3 School of Mechanical and Electrical Engineering, Yunnan Open University, Kunming 650101, China

**Keywords:** polylactic acid, metal–organic framework (MIL-68), mixed-matrix membrane, water treatment, adsorption–filtration, p-nitrophenol

## Abstract

The complete removal of persistent aromatic organic pollutants from aqueous environments demands the development of sustainable and highly efficient filtration materials. In this study, novel bio-sourced mixed-matrix membranes (MMMs) were successfully fabricated by incorporating the highly porous metal–organic framework MIL-68(Al) into a biodegradable polylactic acid (PLA) matrix via a solvent-induced phase inversion method. The integration of MIL-68(Al) nanoparticles significantly tailored the membrane’s morphological structure, endowing the hybrid membranes with enhanced surface hydrophilicity (water contact angle reduced from 90.3° to 72.7°) and superior permeability. The pure water flux reached an optimal value of 42.2 L m^−2^ h^−1^ at a 15 wt.% MOF loading. Crucially, the hybrid membranes exhibited exceptionally high adsorptive removal performance for p-nitrophenol (PNP) and methylene blue (MB). Driven by the abundant accessible active sites of the MOF filler, the MIL-20/PLA membrane achieved a maximum equilibrium adsorption capacity of 121.03 μg/cm^2^ (36.90 mg/g) for PNP, representing a remarkable 25.7-fold enhancement over the pristine PLA membrane. Kinetic analyses confirmed that the adsorption process is strictly governed by pseudo-second-order kinetics, indicating a chemisorption mechanism dominated by hydrogen bonding and π–π stacking interactions. Furthermore, the optimized membranes demonstrated outstanding dynamic filtration efficiencies (>80%) and robust regenerability over multiple continuous operating cycles. This work not only highlights the synergistic interfacial effects between MOFs and biodegradable polymers but also provides a highly effective, eco-friendly, and sustainable membrane platform for the advanced remediation of organic-contaminated wastewater.

## 1. Introduction

Water is the cornerstone of human survival and ecosystem stability [1]. However, with the acceleration of global industrialization and urbanization, the continuous discharge of organic and inorganic pollutants has led to a progressive deterioration of the aqueous environment [2]. Among these contaminants, aromatic organic pollutants such as p-nitrophenol (PNP) and methylene blue (MB) are characterized by their high toxicity, strong biological persistence, and tendency to bioaccumulate through the food chain, posing severe threats to both human health and ecological integrity [3,4]. Consequently, the development of efficient, rapid, and sustainable technologies for the removal of these persistent organic pollutants from wastewater has become a global priority in environmental remediation.

Compared to traditional chemical treatment methods—such as coagulation, chemical precipitation, and advanced oxidation, which often suffer from high operational costs and the generation of toxic secondary by-products—membrane separation technology offers a sustainable and “green” alternative [5]. Membrane processes are highly valued for their high separation precision, energy efficiency, small footprint, and operational simplicity [6]. Unlike chemical reactions that are often irreversible and difficult to control, membrane separation does not involve phase changes and can be precisely modulated based on pressure differentials [7]. In the context of the global transition toward a low-carbon economy, there is an increasing demand for high-performance membrane materials fabricated from environmentally friendly and sustainable resources [8].

Polylactic acid (PLA) is a prominent bio-sourced and biodegradable aliphatic polyester derived from renewable resources, exhibiting excellent biocompatibility, thermal resistance, and mechanical processability [9,10]. While traditional petroleum-based polymers (e.g., PVDF, PES) dominate the current commercial membrane market, their resistance to natural degradation significantly contributes to the escalating issue of secondary microplastic pollution in aquatic systems. PLA presents a highly viable sustainable substitute for these conventional plastics [9,11]. However, its practical application in wastewater treatment is frequently hindered by its intrinsic strong hydrophobicity, low water permeance, and a critical deficiency of active functional groups necessary for specific pollutant binding. Integrating functional nano-fillers into the PLA matrix to construct mixed-matrix membranes (MMMs) has emerged as an effective strategy to synergistically enhance both the permeability and targeted adsorption capacity of the pristine polymer [9].

Metal–Organic Frameworks (MOFs) have attracted significant attention as advanced porous fillers for MMMs due to their exceptionally high specific surface areas, tunable pore sizes, and abundant catalytic or adsorption active sites [12,13]. Among the Materials Institute Lavoisier (MIL) series, MIL-68(Al)—comprising infinite chains of AlO_4_(OH)_2_ octahedra linked by terephthalate ligands—stands out for its remarkable structural robustness and chemical stability in air, aqueous solutions, and mildly alkaline environments [14,15]. Previous studies have demonstrated that the unique Kagomé-like topology of MIL-68(Al) provides highly accessible channels for the rapid diffusion and capture of small-molecule pollutants via hydrogen bonding, electrostatic attraction, and π–π stacking interactions [16,17,18]. While MIL-68(Al) has been incorporated into PVDF and other polymers [19,20], exploring its synergistic combination with a fully biodegradable PLA matrix remains a highly promising yet underexplored frontier.

In recent years, an increasing number of studies have explored the integration of functional fillers into polymeric matrices for the remediation of organic pollutants. Traditional petroleum-based mixed-matrix membranes (MMMs), such as UiO-66-NH_2_/PVDF and UiO-66-NH_2_/PES, typically exhibit notable pure water fluxes (e.g., up to 231.6 L m^−2^ h^−1^) and significant separation capacities [21,22]. However, their non-biodegradable nature poses a severe risk of secondary microplastic pollution in aquatic ecosystems, and their excessively high tensile strength is often over-engineered for low-pressure or gravity-driven wastewater treatment scenarios. In contrast, emerging bio-based materials, particularly polylactic acid (PLA), have demonstrated superior eco-friendliness and sustainability. For instance, a recently developed 3D-printed PLA structure impregnated with graphene oxide exhibited an excellent adsorption capacity of 25.9 mg/g for pharmaceutical pollutants [23]. Yet, to the best of our knowledge, the fabrication of functional MMMs utilizing PLA as the continuous matrix and MOFs as fillers remains exceedingly rare and underexplored. Bridging this significant research gap is highly desirable to develop next-generation membrane platforms that combine complete biodegradability with exceptional separation performance.

Driven by this motivation, novel PLA/MIL-68(Al) hybrid membranes were successfully fabricated in this study using a physical blending and solvent-induced phase inversion (NIPS) method. By incorporating highly porous MIL-68(Al) nanoparticles into the PLA matrix, we aim to concurrently overcome the intrinsic hydrophobic limitations of pure PLA membranes and construct an integrated adsorption–filtration system. This work systematically investigates the influence of MIL-68(Al) loading on morphological evolution, mechanical resilience, surface hydrophilicity, and pure water permeance. By optimizing the MOF loading, our hybrid membranes not only achieve highly competitive adsorption capacities towards p-nitrophenol (PNP) and methylene blue (MB), but also deliver a satisfactory pure water flux (42.2 L m^−2^ h^−1^) and adequate mechanical integrity (0.7–1.3 MPa) for low-pressure applications. Furthermore, the batch adsorption mechanisms and dynamic filtration capabilities were comprehensively evaluated to assess the profound potential of these PLA/MIL-68(Al) MMMs as a sustainable, eco-friendly, and practically viable solution for organic wastewater remediation.

## 2. Experimental

### 2.1. Materials

Aluminium chloride hexahydrate (AlCl_3_·6H_2_O, 98%), terephthalic acid (H2BDC, 99%), 1,4-dioxane (DX, 99.9%), anhydrous methanol (MeOH), N-methylpyrrolidone (NMP, 99.5%), and methylene blue (MB) were purchased from Adamas Reagent Co., Ltd. (Shanghai, China). N,N-dimethylformamide (DMF, 99.5%) was obtained from Chron Chemical Co., Ltd. (Chengdu, China). p-Nitrophenol (PNP, 99%) was sourced from Dr. Ehrenstorfer GmbH (Augsburg, Germany). Polylactic acid (PLA, grade 6100D, Mw = 130,000) was supplied by NatureWorks (Minnetonka, MN, USA). All chemicals were of analytical grade and used as received without further purification.

### 2.2. Membrane Preparation

#### 2.2.1. Synthesis of MIL-68(Al)

The synthesis and activation of MIL-68(Al) were carried out according to a modified procedure based on the method reported by Han et al. [17]. Specifically, the crystallization time was slightly prolonged from 18 h to 18.5 h to ensure complete precursor coordination and uniform crystal morphology, thereby preventing agglomeration during subsequent membrane casting. Briefly, H2BDC (5 g, 30 mmol) and AlCl_3_·6H_2_O (4.88 g, 20 mmol) were dissolved in DMF (300 mL). The resulting homogeneous solution was transferred into a round-bottom flask equipped with a reflux condenser and heated at 130 °C for 18.5 h under continuous stirring. After cooling to room temperature, the white solid product was collected via centrifugation. To remove unreacted organic linkers and residual DMF within the pores, the product was thoroughly washed with fresh DMF three times, followed by methanol washing three times. Finally, the purified MIL-68(Al) was dried in a vacuum oven at 120 °C for 12 h before use.

#### 2.2.2. Preparation of the MIL-68(Al)/PLA Hybrid Membranes

The MIL-68(Al)/PLA mixed-matrix membranes (MMMs) were fabricated via a physical blending and solvent-induced phase inversion (NIPS) method. A co-solvent system comprising NMP and DX at a 1:1 mass ratio was utilized. The concentration of PLA in the casting solution was fixed at 15 wt.%. To systematically investigate the effect of MOF loading, MIL-68(Al) was incorporated at mass fractions of 0%, 5%, 10%, 15%, and 20% relative to the PLA mass. The corresponding hybrid membranes were denoted as MIL-0/PLA (pristine PLA), MIL-5/PLA, MIL-10/PLA, MIL-15/PLA, and MIL-20/PLA, respectively.

The NMP/DX solvent mixture was deliberately selected because NMP acts as an excellent primary solvent for PLA to ensure uniform MIL-68(Al) dispersion, while the more volatile DX serves as a co-solvent to tune the phase separation kinetics. A brief 10-s air evaporation step prior to immersion in the coagulation bath was strictly controlled. This short duration allows for the partial volatilization of DX from the outermost surface, rapidly inducing the formation of a thin, dense, and defect-free selective skin layer (crucial for pollutant rejection) while preventing excessive densification that would otherwise compromise the pure water flux, ultimately yielding an optimized asymmetric porous morphology.

In a typical procedure for MIL-5/PLA, 3 g of PLA and 150 mg of pre-activated MIL-68(Al) were sequentially added into a flask containing 10 mL NMP and 10 mL DX. The mixture was vigorously stirred at 80 °C for 24 h to ensure complete dissolution and uniform dispersion. Subsequently, the casting solution was degassed via ultrasonication to eliminate trapped air bubbles. The homogeneous solution was then cast onto a clean glass plate using a standard 200 μm film applicator. After a 10-s evaporation period in the air, the glass plate was swiftly immersed into a deionized water coagulation bath to induce phase separation. The detached membrane was transferred to fresh deionized water and soaked for 24 h to completely remove residual solvents. Finally, the hybrid membranes were air-dried at room temperature and then vacuum-dried at 80 °C for 12 h.

**Figure 1:**
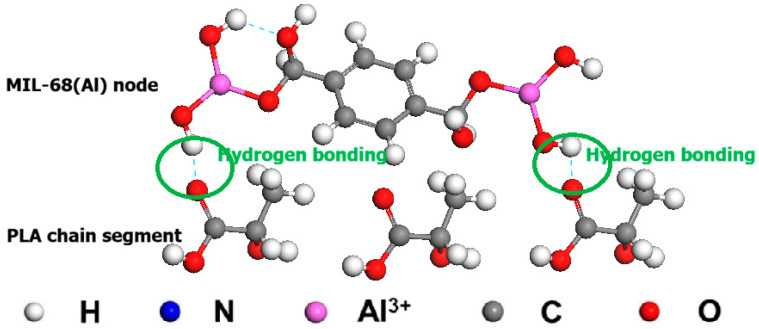
Schematic illustration of the proposed interfacial non-covalent interactions: hydrogen bonding networks formed between the carbonyl oxygen atoms of the PLA chain segments and the bridging hydroxyl groups of the MIL-68(Al) nodes.

To better elucidate the rational design of the hybrid membrane from a polymer chemistry perspective, the chemical structures of the PLA matrix and the MIL-68(Al) filler, along with their proposed non-covalent interfacial interactions, are illustrated in Figure 1. As depicted, the rich bridging hydroxyl groups (Al-OH) and terephthalate linkers intrinsic to the MIL-68(Al) framework provide abundant hydrogen-bond donor/acceptor sites. Concurrently, the extensive carbonyl oxygen atoms (C=O) within the PLA ester bonds act as effective hydrogen-bond acceptors. The resulting strong intermolecular hydrogen bonding across the organic–inorganic interface significantly minimizes interfacial voids, ensuring the excellent compatibility and structural integrity of the mixed-matrix membrane discussed above.

Regarding the stability of these non-covalent interfacial links, while individual hydrogen bonds are relatively weak, the multivalent nature of the abundant interacting sites along the long PLA chains and the vast MOF surface area generates a highly robust, cooperative cross-linking network. Furthermore, the inherent hydrophobicity of the PLA backbone effectively acts as a protective shield, limiting excessive hydration that might otherwise disrupt these interfacial hydrogen bonds in an aqueous environment. The macroscopic stability of these interactions is practically validated by the excellent structural integrity and sustained separation performance of the hybrid membrane during the repeated dynamic filtration–regeneration cycles (as elaborated in Section 3.6), where no severe MOF leaching or performance deterioration was observed under continuous hydrodynamic stress.

### 2.3. Structure Characterization and Membrane Performances Measurement

The morphological structures of MIL-68(Al) and the cross-sections/surfaces of the hybrid membranes were examined using a Field Emission Scanning Electron Microscope (NOVA NANOSEM-450, FEI, Hillsboro, OR, USA) operated at 5 kV, following gold sputter-coating. The crystalline structures were identified via X-ray diffraction (XRD, Bruker D8 ADVANCE A25X, Karlsruhe, Germany) with a scanning rate of 5° min^−1^ from 4° to 40°. Fourier Transform Infrared (FT-IR) spectra were recorded on a Nicolet iS10 spectrometer (Thermo Fisher Scientific, Waltham, MA, USA) using the KBr pellet technique.

The specific surface area and pore size distribution of MIL-68(Al) were determined through N2 adsorption–desorption isotherms at 77 K using a surface area analyzer (BELSORP-MAX, MicrotracBEL, Osaka, Japan). Prior to the measurements, the synthesized MIL-68(Al) powder was degassed under high vacuum at 120 °C for 12 h to completely remove any trapped guest molecules or moisture within the pores. The specific surface area was calculated based on the Brunauer–Emmett–Teller (BET) method using data within the appropriate relative pressure (p/p_0_) range of 0.05 to 0.20. The pore size distribution was evaluated using the Non-Local Density Functional Theory (NLDFT) model, which is highly suitable for accurately assessing the microporous nature of the MOF.

Mechanical properties were evaluated using an Electronic Universal Testing Machine (CMT4104, MTS Systems). Water contact angles were measured using a Theta Lite device (Biolin Scientific, Gothenburg, Sweden) to assess surface hydrophilicity.

The porosity of the films was calculated based on their dry and wet weights. The wet film was weighed after soaking in water for 24 h and wiping off the surface water, and the dry film was weighed after drying at 80 °C for 24 h. The porosity of the film (*δ*) was calculated using Equation (1):

(1) $$\delta =\frac{(\omega_{1}-\omega_{2})/\rho_{w}}{(\omega_{1}-\omega_{2})/\rho_{w}+\omega_{2}/\rho_{p}}\times 100\%$$

where *ω*_1_ and *ω*_2_ represent the weights of the wet and dry films, respectively, and $\rho_{w}$ and $\rho_{p}$ are the densities of water (0.998 g/cm^3^) and PLA (1.24 g/cm^3^), respectively.

The pure water flux (PWF, $J_{w}$) was evaluated using a dead-end ultrafiltration cell (MSC050, Mosu, Hong Kong, China). Membranes with an effective area of 11.34 × 10^−4^ m^2^ were pre-compacted at 0.15 MPa, and the steady flux was recorded at 0.1 MPa over 30 min using Equation (2).

(2) $$J_{w}=\frac{V_{w}}{A\times t}$$

In this context, *V_w_* represents the volume of water that has permeated through the membrane, measured in liters (L), A denotes the effective area of the membrane, calculated to be 11.34 × 10^−4^ m^2^ with an effective diameter of 38 mm, and t corresponds to the duration of the experiment, expressed in hours (h).

### 2.4. Adsorption Experiments

Batch adsorption experiments were conducted to evaluate the removal efficiency for PNP and MB. Membrane samples (2.5 cm × 2.5 cm) were immersed in 10 mL pollutant solutions. Dynamic filtration experiments were conducted using a dead-end filtration setup under a constant transmembrane pressure of 0.1 MPa. In terms of flow configuration, the hybrid membrane was firmly secured in the filtration cell with its active separation layer (the dense surface formed during phase inversion) facing directly towards the feed solution. To minimize concentration polarization near the membrane surface, the feed solution was continuously agitated using a magnetic stirrer.

The initial pH was adjusted using 0.1 M HNO_3_ or 0.1 M NaOH. The residual concentrations of MB and PNP were quantified using a UV-Vis spectrophotometer at 664 nm and 318 nm, respectively. The adsorption capacity ($Q_{e}$) and removal efficiency (η) were calculated using Equations (3) and (4).

(3) $$Q_{e}=\frac{(c_{0}-c_{1})\times V}{S}$$

(4) $$\eta =\frac{c_{0}-c_{1}}{c_{0}}\times 100\%$$

Here, *c*_0_ and *c*_1_ (in mg/L) represent the initial and final concentrations of the pollutants, respectively, *V* (in L) denotes the volume of the pollutant solution, and *S* (in cm^2^) indicates the membrane’s surface area.

To further elucidate the adsorption mechanisms, the time-dependent adsorption data were fitted using the pseudo-first-order and pseudo-second-order kinetic models, which are expressed as Equations (5) and (6), respectively:

(5) $$q_{t}=Q_{e}(1-e^{-k_{1}t})$$

(6) $$q_{t}=\frac{k_{2}Q_{e}^{2}t}{1+k_{2}Q_{e}t}$$

Here, *Q_e_* and *q_t_* (in μg cm^−2^) represent the adsorption amounts at equilibrium and at time *t* (minutes), respectively; *k*_1_ (in min^−1^) and *k*_2_ (in cm^2^ μg^−1^min^−1^) are the rate constants for pseudo-first-order and pseudo-second-order adsorption, respectively.

Furthermore, to elucidate the diffusion mechanisms and identify the rate-limiting steps during the adsorption process, the kinetic data were further analyzed using the Weber–Morris intra-particle diffusion model, expressed as Equation (7):

(7) $$q_{t}=k_{ip}t^{1/2}+C$$

where $k_{ip}$ (μg cm^−2^ min^−1/2^) is the intra-particle diffusion rate constant, and *C* (μg cm^−2^) is a constant related to the boundary layer thickness.

For reusability tests, the pollutant-loaded membranes were thoroughly desorbed by rinsing with ethanol three times, followed by vacuum drying at 60 °C (333 K) for 12 h before the subsequent adsorption cycle. Dynamic filtration experiments were carried out using a silica sand filtration setup under a constant pressure of 0.1 MPa.

This experimental procedure was repeated ten times to elucidate the relationship between the removal efficiency (%) and the incremental filtration volume.

## 3. Results and Discussion

### 3.1. Characterisation of MIL-68(Al)

**Figure 2:**
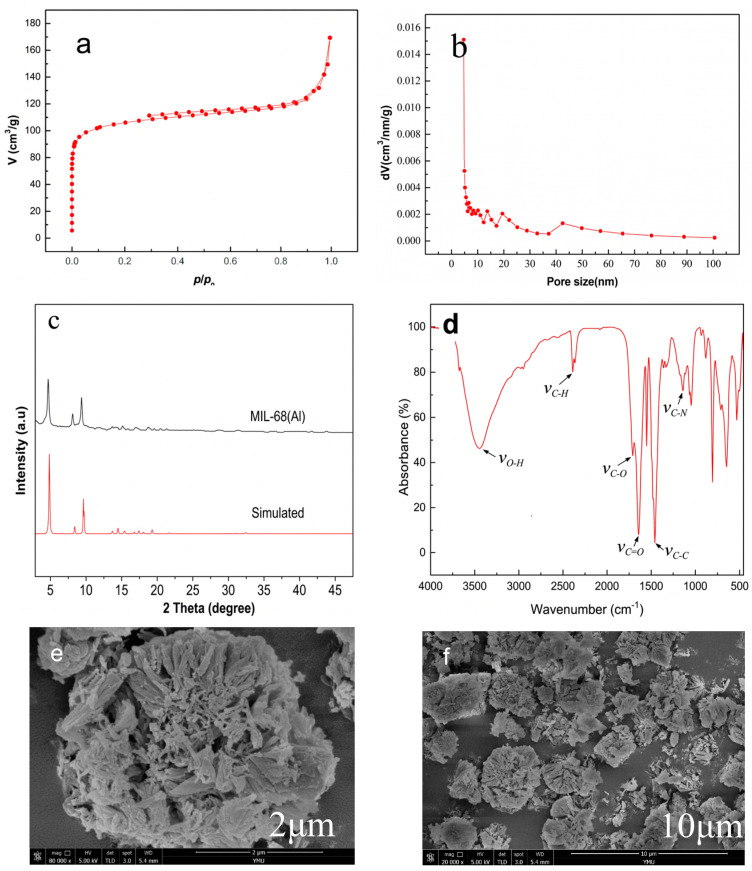
(**a**) N_2_ adsorption isotherm, (**b**) pore size distribution, (**c**) XRD pattern, (**d**) FTIR spectrum, and (**e**,**f**) SEM images of MIL-68(Al).

The successful synthesis of MIL-68(Al) was confirmed through comprehensive characterization. As shown in Figure 2a, the N_2_ adsorption–desorption isotherm exhibits a typical Type I curve, indicating the predominantly microporous nature of the synthesized MOF. The Brunauer–Emmett–Teller (BET) specific surface area reached 1278 m^2^/g with a total pore volume of 0.66 cm^3^/g, providing abundant active sites for pollutant capture. The pore size distribution (Figure 2b), calculated based on the NLDFT method, confirms that the pore structure is predominantly concentrated in the microporous region (<2 nm, according to the IUPAC classification), with a minor extension into the small mesoporous range up to 2.2 nm. The XRD pattern (Figure 2c) displays distinct characteristic diffraction peaks at 2θ values of approximately 5°, 10°, and 15°, which precisely match the simulated pattern of the MIL-68 topology, confirming its high crystallinity. The FT-IR spectrum (Figure 2d) reveals the characteristic vibrations of the terephthalate linkers and Al-O bonds, with the peak at 3675 cm^−1^ ascribed to the structural μ4-OH groups. SEM imagery (Figure 2e,f) illustrates the typical rod-like crystal morphology of MIL-68(Al), confirming its successful preparation.

### 3.2. Characterisation of the Hybrid Membrane

#### 3.2.1. Morphology (SEM)

**Figure 3:**
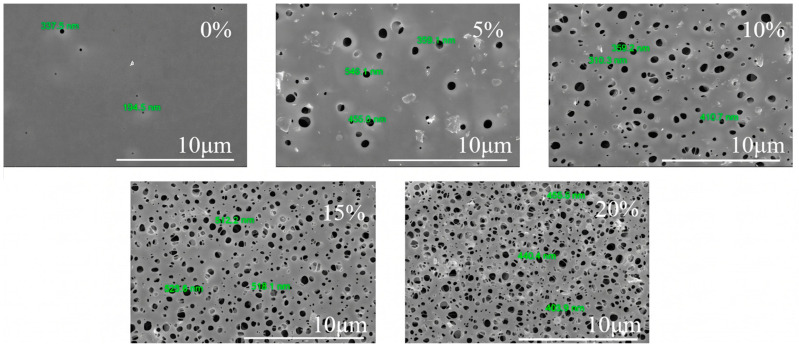
SEM images of the surface of MIL-68 (Al)/PLA hybrid films with different MIL-68(Al) content.

**Figure 4:**
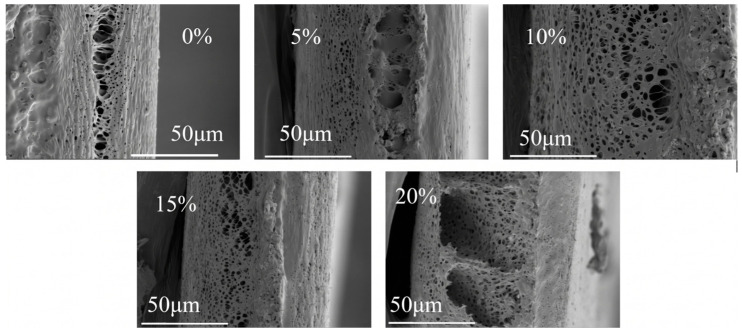
Cross-sectional SEM images of MIL-68 (Al)/PLA hybrid films with different MIL-68(Al) content.

The surface and cross-sectional morphologies of the MIL-68(Al)/PLA hybrid membranes were highly dependent on the MOF loading (Figure 3 and Figure 4). The pristine PLA membrane (MIL-0/PLA) exhibited a relatively dense surface with sparse micropores. Upon the incorporation of MIL-68(Al), the surface pore density increased significantly. This structural evolution can be primarily attributed to the role of hydrophilic MIL-68(Al) nanoparticles acting as heterogeneous nucleation sites during the solvent-induced phase separation process. The presence of these hydrophilic nanoparticles accelerates the mass transfer exchange rate between the solvent (NMP/DX) and the non-solvent (water), leading to a highly porous and interconnected network. However, when the MOF content reached 20 wt.%, particle agglomeration became observable on the membrane surface, which could potentially block some surface pores and affect structural integrity.

**Figure 5:**
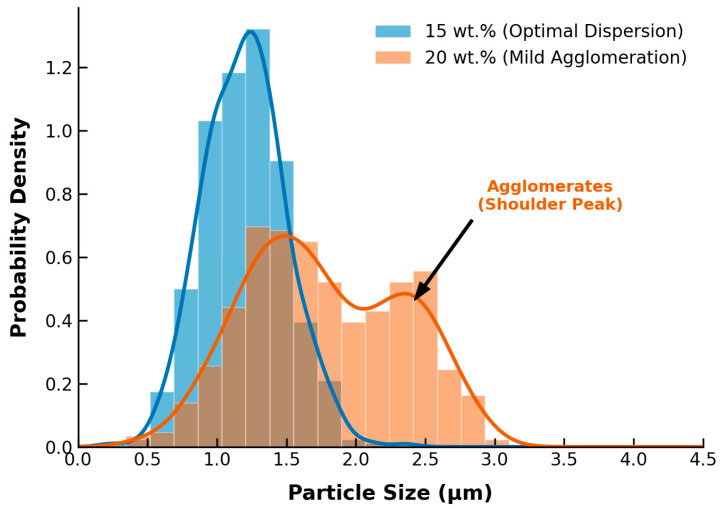
Quantitative particle size distribution of MIL-68(Al) nanoparticles within the PLA matrix at 15 wt.% (optimal dispersion) and 20 wt.% (agglomeration) loadings, derived from cross-sectional SEM image analysis using ImageJ.

To quantitatively verify this dispersion state, the particle size distribution was analyzed using ImageJ software (version 1.53, National Institutes of Health, USA) based on these cross-sectional images (Figure 5). At 15 wt.% loading, the MIL-68(Al) particles exhibited a narrow and uniform size distribution with a primary peak centered around 1.25 µm, further confirming the optimal dispersion. However, visual inspection of the 20 wt.% loaded membrane (Figure 4) reveals noticeable macroscopic particle clusters. When quantitatively analyzed in Figure 5, the corresponding distribution notably broadened and displayed a distinct bimodal characteristic. A secondary shoulder peak emerged at approximately 2.4–2.5 µm, with a significant fraction of particles exceeding 3.0 µm. This quantitative right-shift and the appearance of the secondary peak provide direct mathematical evidence for the agglomeration of MIL-68(Al) nanoparticles at excessive loadings. Consequently, this severe agglomeration reduces the effective specific surface area and creates non-selective voids, which directly leads to the observed decline in pure water flux and mechanical resilience.

#### 3.2.2. Mechanical Properties

The mechanical stability of filtration membranes is crucial for practical applications. As summarized in Table 1, the incorporation of MIL-68(Al) induced a distinct plasticizing and toughening effect on the PLA matrix. The elongation at break significantly increased from 6.0% for pure PLA to a maximum of 39.4% for MIL-15/PLA. This enhancement implies that the finely dispersed MOF nanoparticles disrupt the rigid chain packing of PLA, increasing free volume and allowing better stress dissipation through non-covalent interfacial interactions (e.g., hydrogen bonding). Conversely, the tensile strength gradually decreased from 1.3 MPa to 0.7 MPa with increasing MOF loading. This trade-off is common in MMMs and is generally caused by the inevitable interfacial mismatch and the highly porous structure induced by the MOFs, which act as stress concentration points under load.

Compared to traditional petroleum-based commercial membranes (e.g., PVDF or PES, which typically exhibit tensile strengths > 5 MPa), a strength of 0.7–1.3 MPa is relatively low. However, it is crucial to contextualize this value within real-world application scenarios. The PLA/MIL-68(Al) membranes are specifically targeted for low-pressure or gravity-driven wastewater remediation, where operating pressures typically remain below 0.1 MPa (1 bar). Therefore, a tensile strength of 0.7 MPa (equivalent to roughly 7 bar) provides an adequate structural safety margin for such applications, avoiding the unnecessary over-engineering commonly seen in traditional membranes. Additionally, in practical commercial-scale membrane modules, the active polymeric layer is conventionally cast onto a porous non-woven fabric support to withstand complex hydrodynamic stresses. For future industrial upscaling, this PLA-based active layer could be readily supported by biodegradable non-woven backings to meet more demanding mechanical standards while maintaining its zero-microplastic, eco-friendly footprint.

#### 3.2.3. Hydrophilicity and Permeability

**Figure 6:**
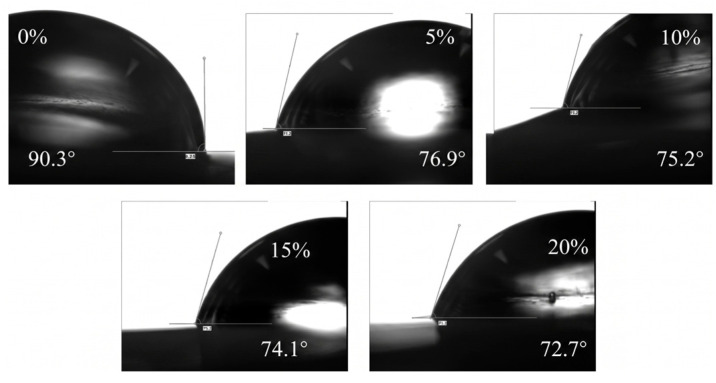
Water contact angle measurements of MIL-68 (Al)/PLA hybrid films with different MIL-68(Al) content.

The wettability of the membranes was evaluated via water contact angle measurements (Figure 6). The pure PLA membrane exhibited a hydrophobic nature with a contact angle of 90.3°. With the step-wise addition of MIL-68(Al) up to 20 wt.%, the contact angle steadily decreased to 72.7°. This substantial improvement in hydrophilicity originates from the inherent hydrophilic functional groups (e.g., -OH, -COOH) present on the MOF surface and the increased surface roughness resulting from enhanced porosity.

**Figure 7:**
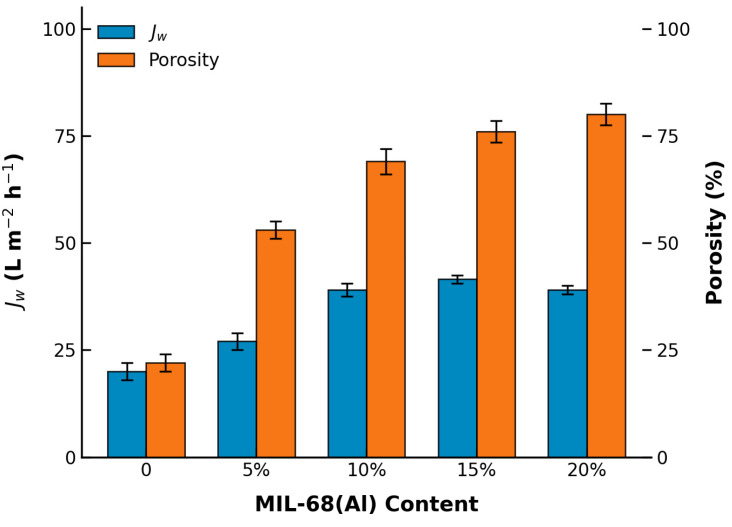
Pure water permeability and porosity of MIL-68 (Al)/PLA hybrid films with different MIL-68(Al) content.

Consequently, the pure water flux (PWF) displayed a strong positive correlation with the MOF loading up to 15 wt.% (Figure 7). The PWF dramatically increased from 18.6 L m^−2^ h^−1^ for pure PLA to a peak value of 42.2 L m^−2^ h^−1^ for MIL-15/PLA, consistent with the increased porosity and improved hydrophilicity observed in SEM and contact angle tests. However, a further increase in MOF loading to 20 wt.% resulted in a slight decline in PWF (38.1 L m^−2^ h^−1^). This flux reduction is likely due to severe nanoparticle aggregation, which congests the internal pore channels and increases hydraulic resistance.

While the optimal pure water flux of 42.2 L m^−2^ h^−1^ is moderate compared to highly permeable, non-biodegradable PVDF-based membranes (e.g., 231.6 L m^−2^ h^−1^ for PVDF/UiO-66-NH_2_) [21], it is highly competitive with, and even distinctly surpasses, several recently reported petroleum-based systems (e.g., 37.8 L m^−2^ h^−1^ for PES/UiO-66-NH_2_) [22]. This moderate but stable flux represents an optimal performance trade-off. We purposefully sacrifice the extreme permeability associated with traditional inert polymers to achieve a 100% eco-friendly, biodegradable PLA matrix with exceptional adsorption capabilities. For targeted low-pressure organic wastewater remediation, this balanced “adsorption–filtration” synergy makes the PLA/MIL-68(Al) approach highly competitive and practically viable.

### 3.3. Adsorption Performance of Hybrid Membranes for PNP and MB

**Figure 8:**
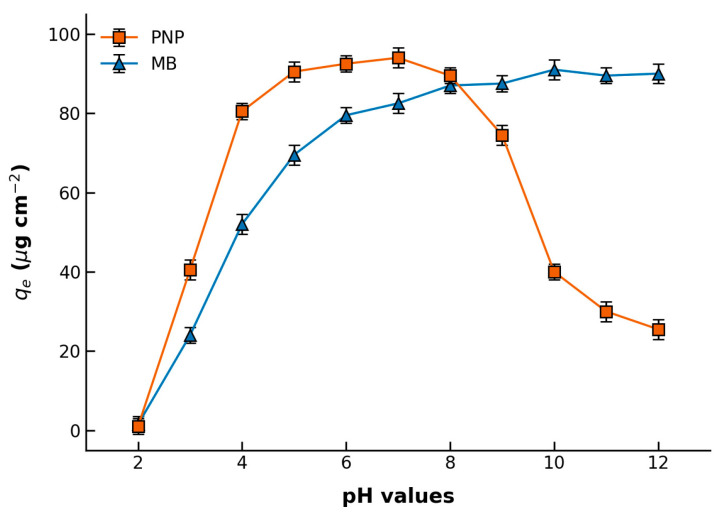
pH-Dependent Adsorption Capacities of PNP and MB on MIL-68(Al)/PLA Hybrid Membrane (the initial concentrations of the two organics are 100 mg/L and 60 mg/L).

pH Dependence: The initial pH of the solution significantly influences both the surface charge of the membrane and the speciation of the pollutants (Figure 8). Although direct streaming zeta potential measurements of the intact membrane surface were constrained by equipment availability, the pH-dependent adsorption mechanism can be robustly elucidated using the well-established point of zero charge ($pH_{pzc}$) of MIL-68(Al) (typically around 5.0–6.0) and the intrinsic negative charge of the PLA matrix in aqueous environments due to its terminal carboxyl groups. At lower pH levels (<4.0–5.0), the partial degradation and surface protonation of the MOF limit both the adsorption capacity for PNP and the electrostatic attraction for the cationic MB. As the solution pH increases beyond 7.0, the hybrid membrane surface becomes increasingly negatively charged. For PNP, optimal adsorption occurs between pH 5.0 and 7.0; however, when the pH exceeds its $pK_{a}$ (~7.15), PNP molecules deprotonate to form phenolate anions. This results in strong electrostatic repulsion with the negatively charged membrane, leading to a sharp decrease in PNP adsorption. Conversely, this highly electronegative membrane surface under alkaline conditions (pH > 8.0) provides a strong driving force for electrostatic attraction, thereby continuously enhancing the adsorption capacity for the cationic dye MB.

**Figure 9:**
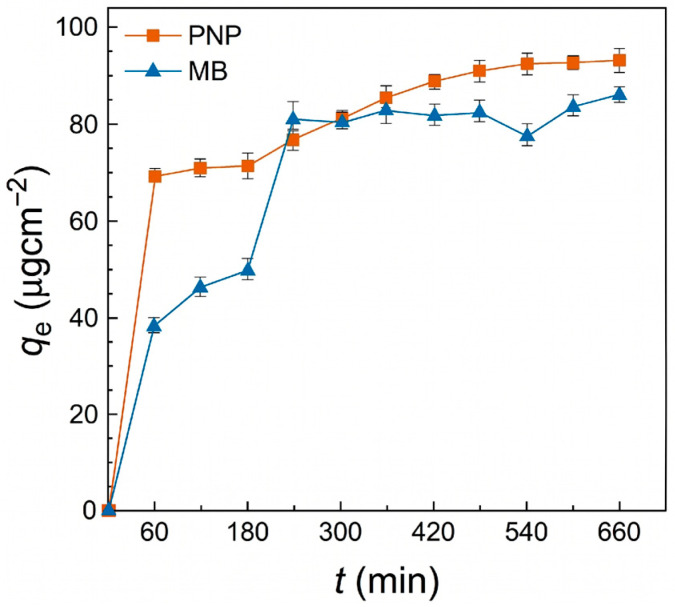
Adsorption kinetics of PNP and MB under the pH of 7.0 with the initial concentrations of 120 mg/L and 60 mg/L respectively.

**Figure 10:**
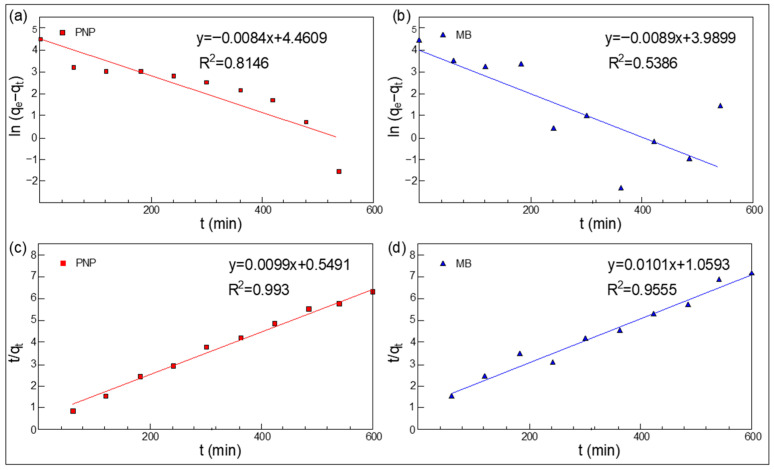
Adsorption kinetics fitting of PNP (**a**,**c**) and MB (**b**,**d**) using pseudo-first-order (**a**,**b**) and pseudo-second-order (**c**,**d**) models.

Adsorption Kinetics: The time-dependent adsorption behavior (Figure 9 and Figure 10) indicates that PNP reaches equilibrium relatively slowly (approx. 540 min), whereas MB adsorption stabilizes earlier. The kinetic data were better described by the pseudo-second-order model for both pollutants (Table 2), particularly for PNP ($R^{2}=0.993$), where the calculated equilibrium capacity ($Q_{e}=101.01$ μg/cm^2^, equivalent to 30.79 mg/g) perfectly matched the experimental value (93.74 μg/cm^2^, 28.58 mg/g). Similarly, the $Q_{e}$ for MB was calculated as 99.01 $\mu g/cm^{2}$ (30.19 mg/g).

The excellent fit of the pseudo-second-order kinetic model strongly indicates that the adsorption of PNP and MB onto the hybrid membranes is primarily governed by a chemisorption process. While direct post-adsorption spectroscopic validations (such as XPS or FTIR shifts) were not conducted in the current study, the specific chemisorption mechanisms can be robustly deduced based on our kinetic data and extensive literature consensus regarding MIL-68(Al). It is widely reported that the characteristic Kagomé topology of MIL-68(Al) provides abundant terephthalate linkers and structural hydroxyl/carboxyl groups. Consequently, the highly efficient capture of aromatic pollutants like PNP and MB is commonly attributed to strong π−π stacking interactions (between the aromatic rings of the pollutants and the organic linkers of the MOF) and hydrogen bonding (involving the electronegative functional groups). This established theoretical framework perfectly corroborates the chemisorption-dominated kinetics observed in our experiments.

**Figure 11:**
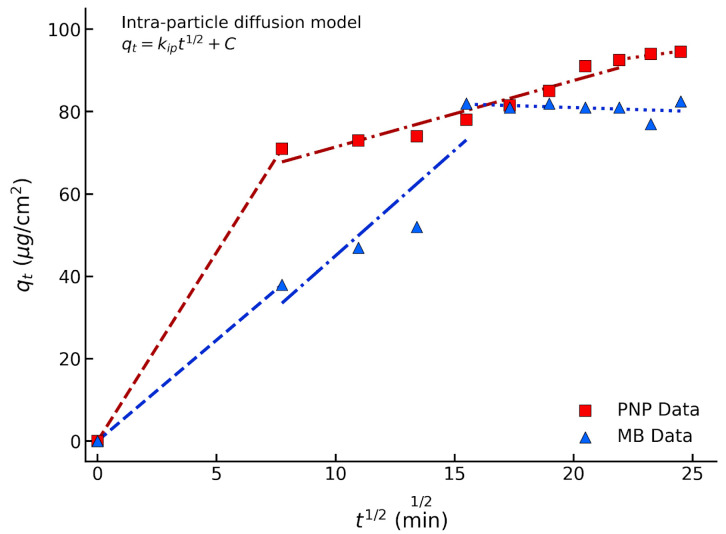
Intra-particle diffusion model plots (*q_t_* versus $t^{1/2}$) for the adsorption of PNP and MB onto the optimal MIL-20/PLA mixed-matrix membrane.

To further investigate the rate-limiting steps, the Weber–Morris intra-particle diffusion model was applied. As shown in the plot of *q_t_* versus *t*^1/2^ (Figure 11), the adsorption process exhibits multi-linear stages rather than a single straight line passing through the origin. The initial steep stage represents the rapid external surface adsorption or film diffusion, where PNP and MB molecules quickly attach to the abundant accessible sites on the membrane surface. The subsequent stage corresponds to the intra-particle diffusion, where the pollutants gradually diffuse deeper into the internal micropores of the MIL-68(Al) framework. The final plateau stage indicates the attainment of adsorption equilibrium. Crucially, the multi-linear nature of the plots and the non-zero intercepts (*C* ≠ 0) demonstrate that intra-particle diffusion is not the sole rate-limiting step; rather, the adsorption kinetics are synergistically governed by external boundary layer diffusion, intra-particle diffusion, and ultimate surface chemisorption.

### 3.4. Effect of MIL-68(Al) Loading on Adsorption Capacity

**Figure 12:**
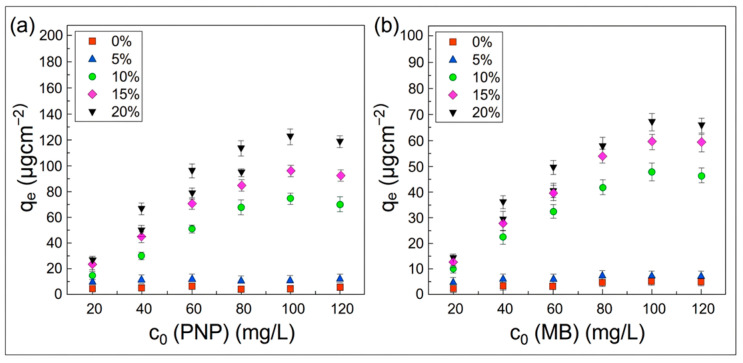
Equilibrium adsorption capacities for PNP (**a**) and MB (**b**) on the pristine PLA and MIL-68(Al)/PLA hybrid membranes with varying MIL-68(Al) loading amounts.

Figure 12 demonstrates the profound impact of MIL-68(Al) incorporation on the equilibrium adsorption capacities for both PNP and MB. As explicitly depicted, the adsorption performance of the hybrid membranes escalates monotonically with the increasing mass fraction of MIL-68(Al). For instance, the adsorption capacity for PNP progressively increases from 15.55 μg/cm^2^ (MIL-5/PLA), to 72.12 μg/cm^2^ (MIL-10/PLA), 96.44 μg/cm^2^ (MIL-15/PLA), and ultimately reaches 121.03 μg/cm^2^ (36.90 mg/g) for MIL-20/PLA. When juxtaposed with the pristine PLA membrane—which exhibits a negligible capacity of merely 4.71 μg/cm^2^—the MIL-20/PLA membrane achieves a remarkable enhancement of approximately 25.7 times. A similar upward trend is observed for MB, with the maximum capacity reaching approximately 28.66 mg/g for the MIL-20/PLA membrane. This massive improvement unambiguously confirms that the embedded MIL-68(Al) nanoparticles act as the primary active adsorption sites, successfully compensating for the intrinsic lack of functional binding sites in the pure PLA matrix.

### 3.5. Dynamic Adsorption Activity

**Figure 13:**
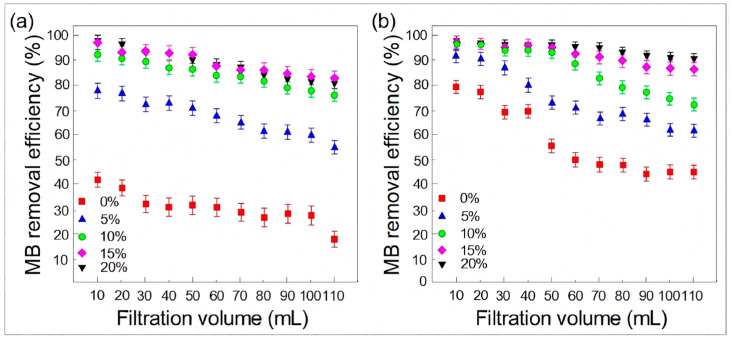
Dynamic adsorption capacities of PNP (**a**) and MB (**b**) by pristine PLA and MIL-68(Al)/PLA hybrid membranes with different adding amounts of MIL-68(Al).

To evaluate practical applicability, dynamic filtration experiments were conducted (Figure 13). The MIL-68(Al)/PLA hybrid membranes exhibited remarkably superior dynamic removal efficiencies compared to pristine PLA. Notably, the MIL-15/PLA and MIL-20/PLA membranes maintained high removal efficiencies of over 80% for PNP and 85% for MB even after processing 100 mL of the feed solution. This outstanding dynamic performance validates the synergistic effect of the hierarchical porous structure (facilitating mass transfer) and the densely exposed MOF active sites (ensuring rapid capture).

### 3.6. Reusability Performances

**Figure 14:**
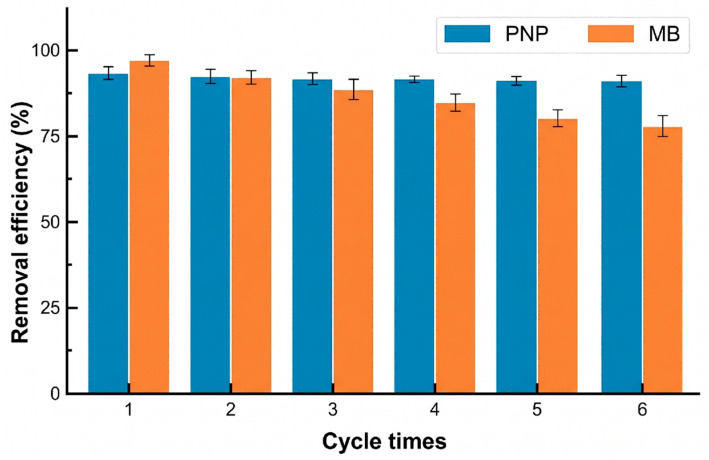
Reusability performance of the optimal MIL-15/PLA mixed-matrix membrane for the removal of PNP and MB across six consecutive adsorption–desorption cycles.

The long-term operational stability of the optimal MIL-15/PLA membrane was evaluated through six consecutive dynamic adsorption–desorption cycles (Figure 14). The membrane demonstrated exceptional and sustained regenerability for PNP, with the removal efficiency merely experiencing a marginal decline from 93.6% in the first cycle to 91.1% in the sixth cycle. This highly stable functional performance serves as strong indirect evidence of the membrane’s structural resilience; if the MIL-68(Al) framework had collapsed or leached out during continuous hydrodynamic and solvent regeneration stress, such sustained high-efficiency chemisorption of PNP would not have been maintained.

For MB, however, the removal efficiency showed a more noticeable decrease (from 97.3% to 77.2% after six cycles). This discrepancy in desorption efficiency is fundamentally ascribed to the distinct physicochemical interactions between the pollutants and the membrane. MB is a relatively large cationic dye (319.85 g/mol) that binds with the highly electronegative PLA/MIL-68(Al) membrane through strong electrostatic attraction under operating conditions. Consequently, simple ethanol washing is insufficient for complete desorption, leading to cumulative micro-pore blockage and the progressive occupation of accessible active sites over successive cycles. In contrast, the smaller PNP molecules (139.11 g/mol) are readily and completely desorbed. Therefore, the observed decline in MB removal is a result of saturation-induced pore blockage rather than the structural degradation of the hybrid membrane.

Regarding the fouling behavior during the dynamic filtration process, it is important to note that the targeted pollutants (PNP and MB) are small aromatic molecules. Therefore, the macroscopic cake-layer fouling typically observed in macromolecular or colloidal filtration was not the primary phenomenon. Instead, the gradual decline in permeate flux and removal efficiency over continuous filtration volumes (and across multiple cycles) was mainly attributed to the adsorptive saturation of the exposed MIL-68(Al) active sites and partial micro-pore blockage by the accumulated dye/organic molecules. This saturation-induced “chemical fouling” proved to be highly reversible, as washing the membrane with ethanol effectively desorbed the trapped molecules, regenerated the active sites, and restored the membrane’s synergistic adsorption–filtration performance.

### 3.7. Practical Applicability and Perspectives

To evaluate the engineering potential of the PLA/MIL-68(Al) hybrid membranes, their practical applicability is assessed in terms of scalability, cost-effectiveness, and comparative advantages over existing commercial technologies.

Firstly, regarding scalability, the solvent-induced phase inversion (NIPS) method employed in this study is the standard, highly mature technology currently used in industrial continuous roll-to-roll membrane manufacturing. The incorporation of MIL-68(Al) nanoparticles involves a simple physical blending step prior to casting, requiring no extreme temperatures, high pressures, or complex in situ growth procedures. Thus, the fabrication process is highly compatible with existing commercial membrane production lines.

Secondly, from a cost perspective, PLA is a commercially mature, bio-sourced polymer whose market price is becoming increasingly competitive with conventional petroleum-based plastics due to the booming global bioplastics industry. Furthermore, the synthesis of MIL-68(Al) utilizes abundant, low-cost aluminum salts and terephthalic acid (H2BDC) as precursors, avoiding the use of expensive precious metals or complex organic ligands, which significantly reduces the filler cost.

Finally, compared to dominant commercial membrane technologies (e.g., PVDF, PES, and PTFE), which primarily rely on physical size-exclusion and suffer from intrinsic bio-inertia, the proposed PLA/MIL-68(Al) MMMs present two unique advantages. On the one hand, the embedded MOFs provide highly specific chemisorption sites for targeted pollutant removal, realizing a dual “adsorption–filtration” function that standard commercial microfiltration/ultrafiltration membranes lack. On the other hand, traditional petroleum-based membranes pose severe challenges for end-of-life disposal and contribute to secondary microplastic pollution in aquatic systems. The complete biodegradability of the PLA matrix offers a sustainable, “green” end-of-life alternative, aligning perfectly with the global transition toward a circular low-carbon economy.

## 4. Conclusions

This study successfully developed biodegradable PLA/MIL-68(Al) mixed-matrix membranes, demonstrating a highly effective synergistic adsorption–filtration paradigm for the removal of aromatic pollutants. By compensating for the inherent hydrophobicity and adsorption deficiency of pristine PLA with the highly porous, functionally rich MIL-68(Al) networks, this work provides a compelling insight into transitioning from non-degradable petroleum-based membranes to sustainable, bio-sourced alternatives without compromising separation performance. The underlying chemisorption mechanism, driven by specific interfacial interactions, highlights the immense potential of MOF–polymer synergy in advanced water remediation.

Despite these promising results, certain limitations must be acknowledged. First, while the hybrid membranes exhibited robust structural stability over multiple laboratory-scale cycles, the long-term degradation kinetics of the PLA matrix and their operational resilience in real, complex wastewater environments remain unexplored. Crucially, the co-existence of diverse background pollutants and natural organic matters in actual wastewater could trigger severe competitive adsorption, potentially leading to much faster pore blockage and a more rapid decline in removal efficiency than observed in our binary model systems. Second, the current proof-of-concept synthesis of MOFs and flat-sheet membranes requires further optimization for cost-effective industrial upscaling. Furthermore, as observed with MB, the strong electrostatic binding of large cationic dyes can lead to irreversible micro-pore blockage, limiting long-term regenerability.

Consequently, future research directions will focus on evaluating these hybrid membranes using real industrial effluents containing competing ions and complex natural organic matters to systematically assess and mitigate competitive pore blockage. Additional efforts will be directed towards exploring continuous pilot-scale module designs (e.g., spiral-wound configurations) and tailoring the membrane surface with anti-fouling modifications to mitigate the irreversible binding of large molecules. Ultimately, this work establishes a robust baseline and theoretical framework for designing next-generation, eco-friendly membrane technologies for sustainable environmental remediation.

## Figures and Tables

**Table 1:** Tensile testing of MIL-68 (Al) /PLA hybrid films with different MIL-68(Al) content.

MIL-68(Al) Content/%	0	5	10	15	20
Elongation at break/%	6.0	18.3	25.8	39.4	36.3
Tensile strength/MPa	1.3	1.0	0.9	0.8	0.7

**Table 2:** Fitting parameters using pseudo-first-order and pseudo-second-order kinetics models for the adsorption kinetics of PNP and MB.

Organics	*q_t(max)_* (μg cm^−2^)	Pseudo-First Order			Pseudo-Second Order		
		*Q_e_* (μg cm^−2^)	*k*_1_ (min^−1^)	*R* ^2^	*Q_e_* (μg cm^−2^)	*k*_1_ (min^−1^)	*R* ^2^
PNP	93.74	86.57	−0.0084	0.8146	101.01	0.0099	0.9930
MB	83.16	54.05	−0.0089	0.5386	99.01	0.0101	0.9555

## Data Availability

The data presented in this study are available on request from the corresponding authors.
